# Supplementary material for: Endothelial RNF20 suppresses endothelial-to-mesenchymal transition and safeguards physiological angiocrine signaling to prevent congenital heart disease
Source: Nat Commun. 2025 Oct 27;16:9480. doi: 10.1038/s41467-025-65291-0 (PMC12559710; doi:10.1038/s41467-025-65291-0)
Supplement: Supplementary file 1 — Supplementary Information [file 41467_2025_65291_MOESM1_ESM.pdf]

Supplementary Information
for

Endothelial RNF20 Suppresses Endothelial-to-Mesenchymal Transition and Safeguards Physiological Angiocrine Signaling to Prevent Congenital Heart Disease

Yanliang Dou[§], Nalan Tetik-Elsherbiny[§], Rui Gao[§], Yonggang Ren, Yu-wen Chen, Moritz Merbecks, Aadhyaa Setya, Olga Lityagina, Yinuo Wang, Evgeny Chichelnitskiy, Aya Abouissa, Chi-Chung Wu, Guillermo Barreto, Michael Potente, Thomas Wieland, Roxana Ola, Philippe Grieshaber, Tsvetomir Loukanov, Matthias Gorenflo, Jörg Heineke, Julio Cordero*, and Gergana Dobreva*

This PDF files includes:

Supplementary Fig. 1 to 10

Supplementary Table 1 to 4

Other online data supplement for this manuscript include the following:

Supplementary Data 1 to 8 (Excel spreadsheets)

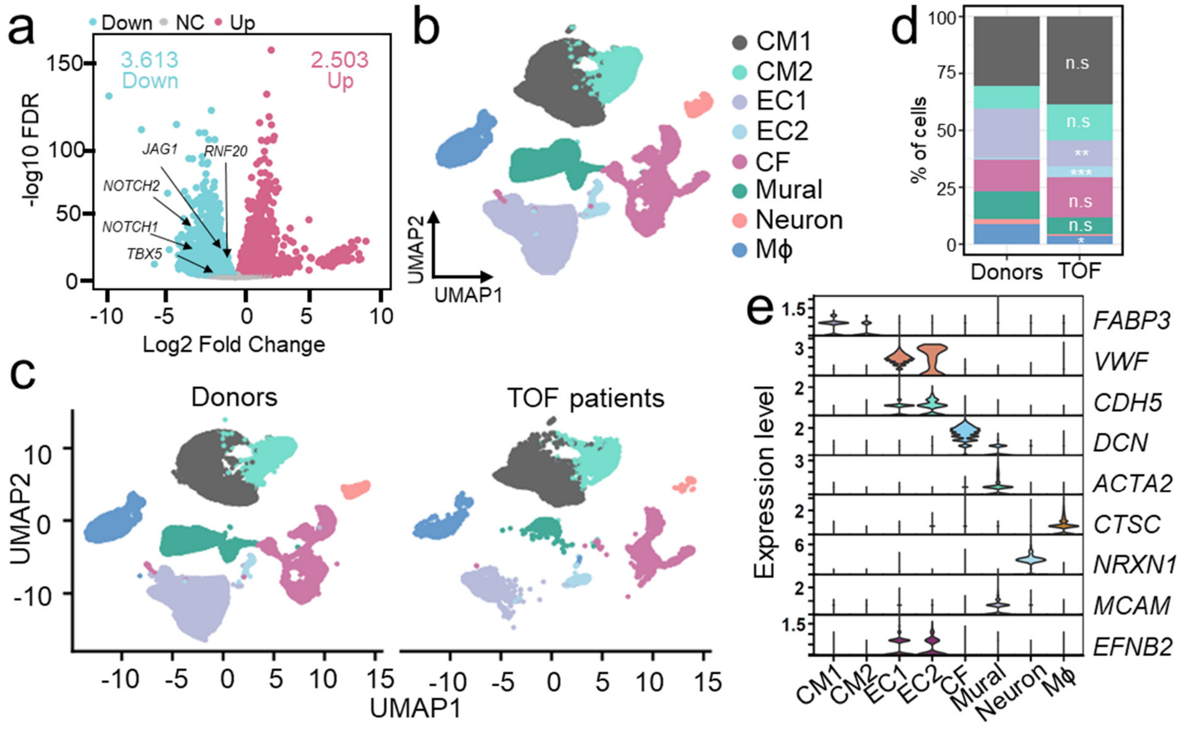

Supplementary Fig. 1. RNF20 expression levels in donor and TOF patients.

a Volcano plot of differentially expressed data taken from [47] (GSE256516). Dots representing *RNF20* as well as CHD-related genes, such as *JAG1*, *NOTCH1*, *NOTCH2*, and *TBX5* are highlighted.

b UMAP of donor and TOF patients from GSE203274, colored by cell type. CM, cardiomyocytes, EC, endothelial cells, CF, cardiac fibroblasts, Mϕ, macrophages.

c UMAP plot grouped by patient category: Donor, representing healthy donor heart samples; and TOF, right ventricular heart samples from patients aged 3–8 months.

d Bar plot showing the percentage of cells within the different clusters colored by cell type. Data are shown as the frequency of the different cardiac cell types in donor and TOF samples. P-values were calculated using a two-sided exact binomial test. Statistical significance is indicated as follows: n.s., not significant ($p > 0.05$); *$p < 0.05$; **$p < 0.01$; *$p < 0.001$.

e Violin plot displaying expression levels of selected marker genes across eight annotated cell types.

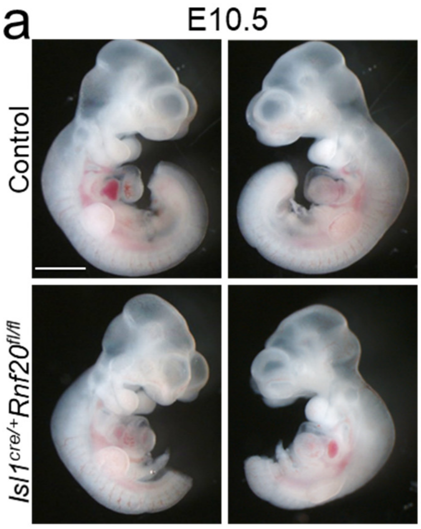

b

	Ao/PA	Single vessel	Total
Control (WT)	6	0	6
Isl1$^{cre/+}$*Rnf20*$^{fl/fl}$	0	5	5
Total	6	5	11
Fisher's exact test			p = 0.0022

c

	Arrhythmic	Normal Rhythm	Total
Control (WT)	1	11	12
Isl1$^{cre/+}$*Rnf20*$^{fl/fl}$	5	1	6
Total	6	12	18
Fisher's exact test			p = 0.0039

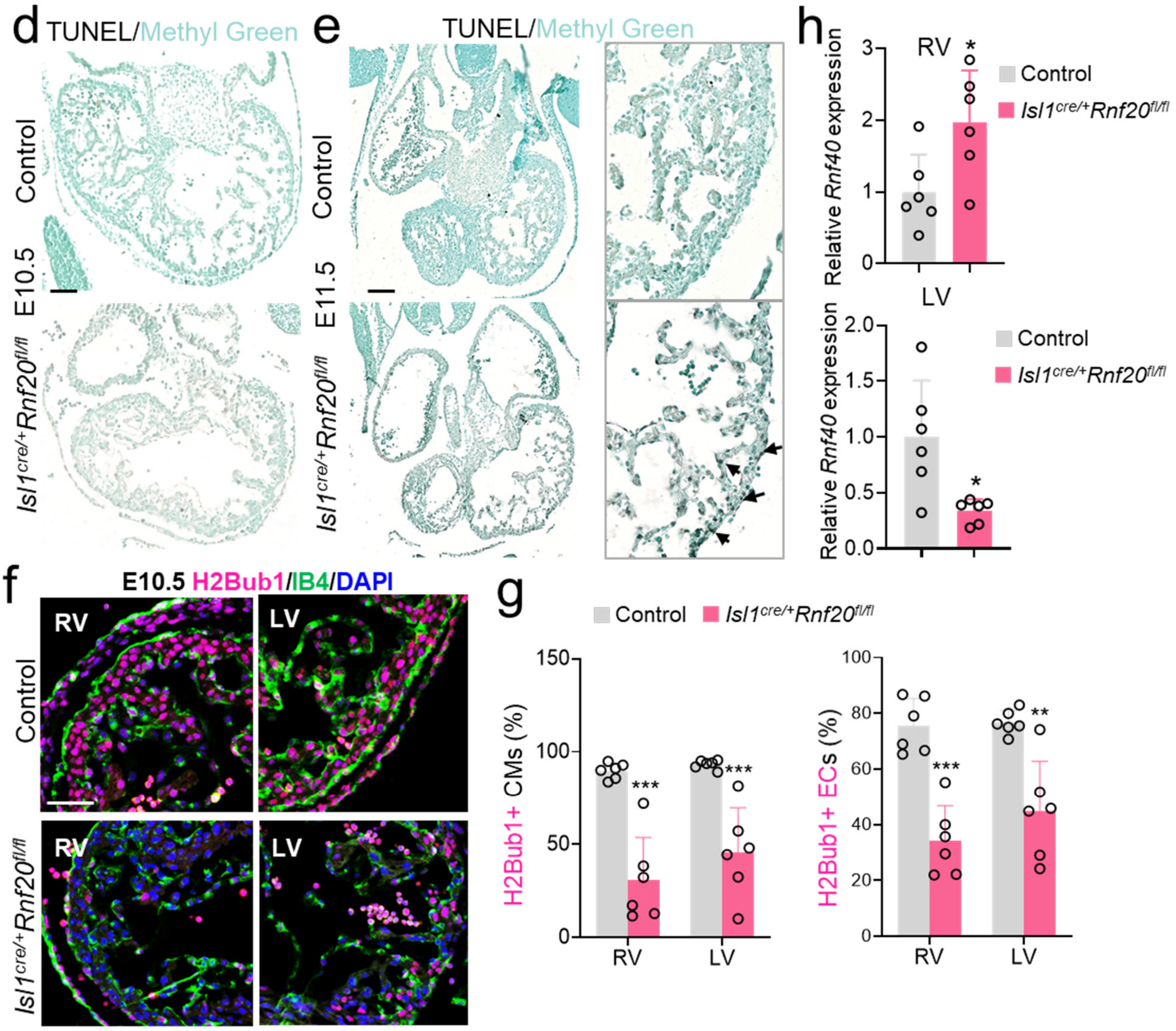

Supplementary Fig. 2. Function of RNF20 in SHF Development.

a Gross appearance of control and *Isl1*$^{cre/+}$*Rnf20*$^{fl/fl}$ embryos at E10.5. Scale bars, 1 mm.

b Contingency table showing the incidence of single vessel in control and *Isl1*$^{cre/+}$*Rnf20*$^{fl/fl}$ hearts. Statistical significance was assessed using Fisher's exact test ($p = 0.0022$).

c Contingency table showing the incidence of arrhythmic behavior in control and *Isl1*$^{cre/+}$*Rnf20*$^{fl/fl}$ hearts. Statistical significance was assessed using Fisher's exact test ($p = 0.0039$).

d, e TUNEL analysis to detected apoptotic cells on sections of control and *Isl1*$^{cre/+}$*Rnf20*$^{fl/fl}$ E10.5 **(d)** and E11.5 **(e)** embryos. Nicked DNA was visualized by HRP staining. Methyl green staining was used to visualize nuclei.

f, g Immunostaining for H2Bub1, catalyzed by RNF20, MF20 and anti-IB4 of E10.5 control (n=6) and *Isl1*$^{cre/+}$*Rnf20*$^{fl/fl}$ (n=6) hearts (**f**) and quantification of H2Bub1-positive CMs (H2Bub1+/MF20+ cells) and H2Bub1-positive ECs (H2Bub1+/IB4+ cells) in the LV and the RV (**g**). Scale bars in **f**, 50 µm.

h Relative *Rnf40* mRNA expression in RV and LV of E10.5 control (n=6) and *Isl1*$^{cre/+}$*Rnf20*$^{fl/fl}$ (n=6) hearts.

Statistical analysis between two groups in **g**, **h**, was performed using an unpaired two-tailed Student's t-test. Data are shown as means ± SEM. $*p<0.05$, $**p<0.01$, $***p<0.001$.

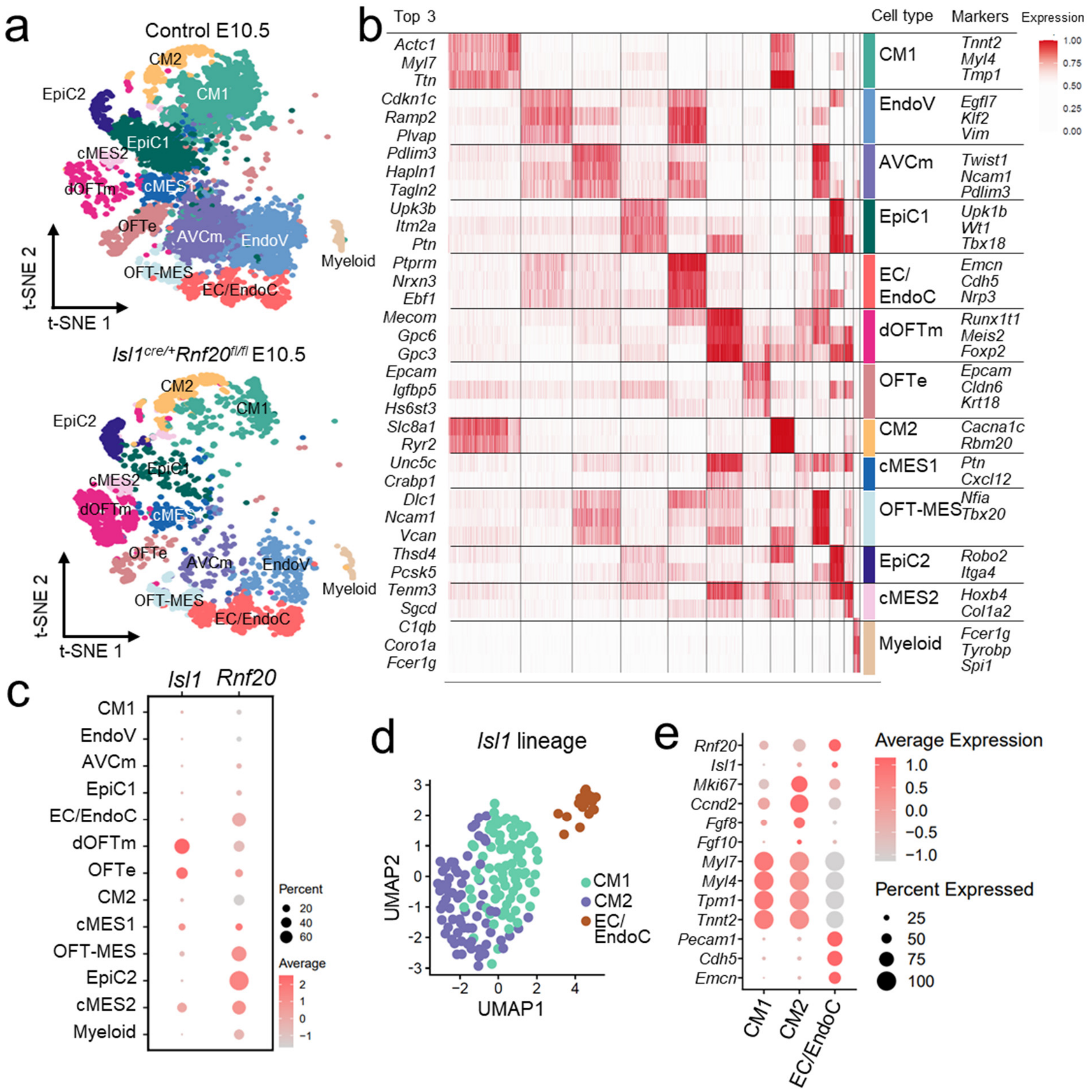

Supplementary Fig. 3. Single cell analysis of control and *Isl1*$^{cre/+}$*Rnf20*$^{fl/fl}$ hearts.

a t-SNE plot separated by genotype and colored according to cell type.

b Heatmap showing the top 3 markers for each cell type on the left, and markers used to define the cell populations from Fig. 3b on the right.

c Dot plot illustrating the expression of *Isl1* and *Rnf20* across various cell types. Size of the dots reflects the percentage of cells expressing *Isl1* or *Rnf20* and color represents the average expression level in the different cell types in control (n=5) and *Isl1*$^{cre/+}$*Rnf20*$^{fl/fl}$ (n=5) E10.5 hearts.

d UMAP plot depicting cell clusters within the *Isl1* lineage in E8.75 embryos[49].

e Dot plot displaying marker genes for CMs, ECs, and proliferative populations. Dot size indicates the percentage of cells expressing *Isl1*, *Rnf20*, and the respective marker genes, while color represents the average expression level.

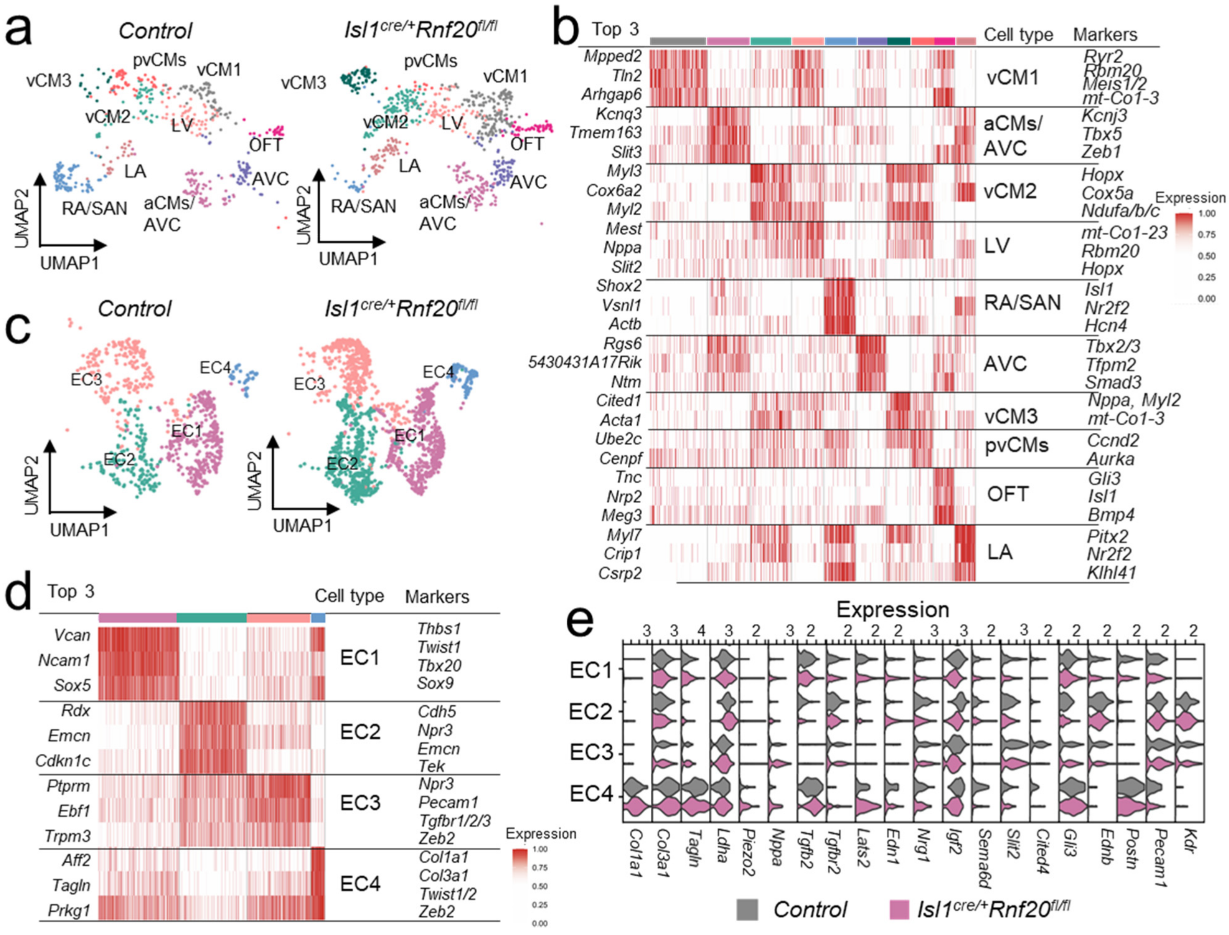

Supplementary Fig. 4. Cardiomyocyte and endothelial clusters in control and *Isl1$^{cre/+}$Rnf20$^{fl/fl}$* hearts.

a t-SNE plot separated by genotype and colored according to CM subtype. **b** Heatmap showing the top 3 markers for each CM subtype on the left, and markers used to define the cell populations from Fig. 3f on the right.

c t-SNE plot separated by genotype and colored according to EC subtype. **d** Heatmap showing the top 3 markers for each EC subtype on the left, and markers used to define the cell populations from Fig. 3h on the right.

e Violin plots visualizing expression levels of selected up- and downregulated genes in the different EC populations in control and *Isl1$^{cre/+}$Rnf20$^{fl/fl}$* hearts.

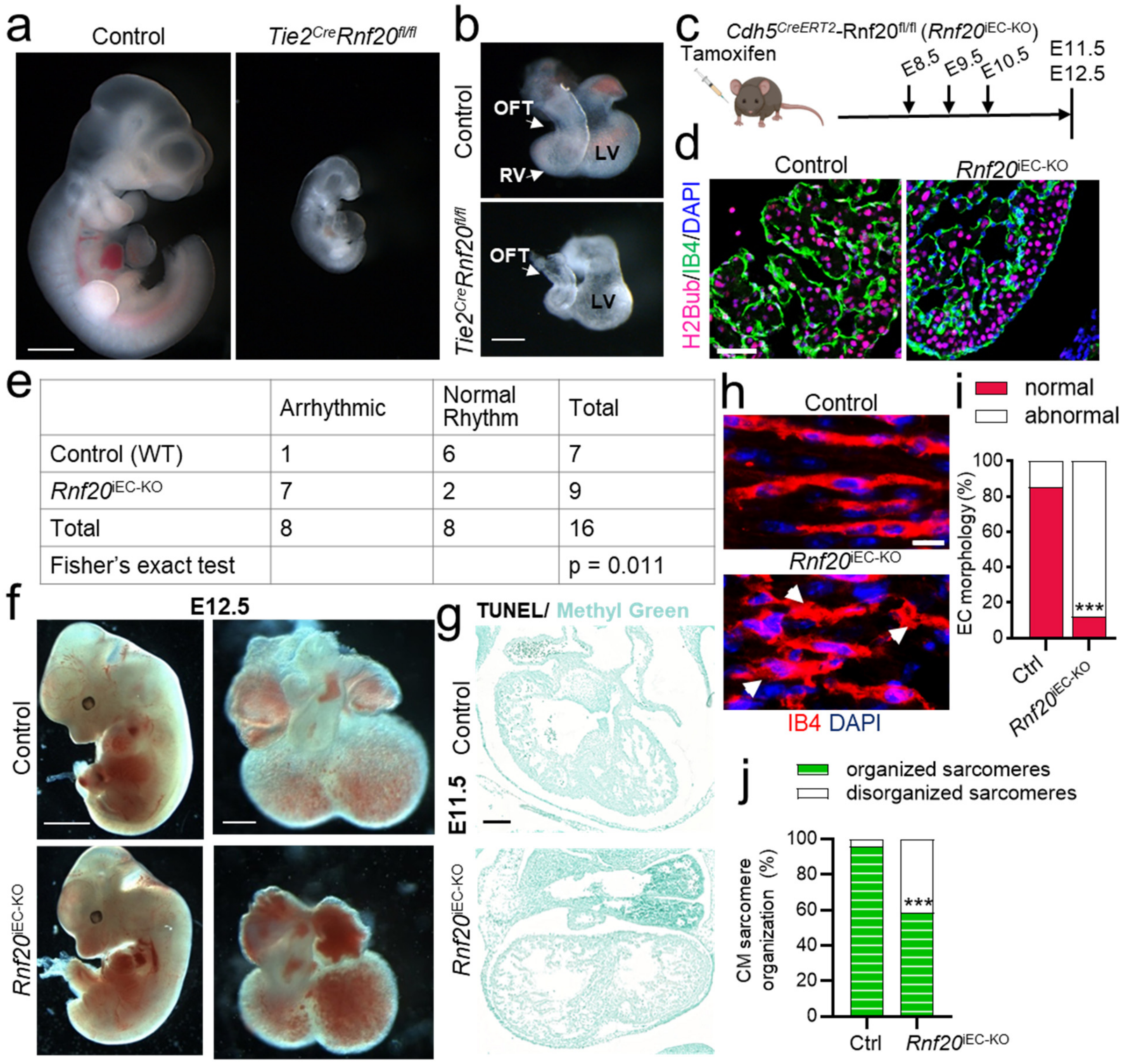

	Arrhythmic	Normal Rhythm	Total
Control (WT)	1	6	7
Rnf20$^{iEC\text{-}KO}$	7	2	9
Total	8	8	16
Fisher's exact test			p = 0.011

Supplementary Fig. 5. Function of endothelial RNF20 in cardiac development.

a, b Gross appearance of control (*Tie2*^{Cre}*Rnf20*$^{+/fl}$) and *Tie2*^{Cre}*Rnf20*$^{fl/fl}$ embryos at E10.5 (**a**) and of dissected hearts (**b**). Scale bars in a, 1 mm. Scale bars in b, 200 µm.

c Schematic representation of the experimental setup.

d Immunostainings of H2Bub1 in control and *Rnf20*$^{iEC\text{-}KO}$ hearts at E11.5 (bottom), showing loss of Rnf20 specifically in endothelial cells. For this experiment and all other studies, control (*Cdh5*^{CreERT2}neg-*Rnf20*$^{fl/fl}$) and *Rnf20*$^{iEC\text{-}KO}$ (*Cdh5*^{CreERT2}pos-*Rnf20*$^{fl/fl}$) embryos were exposed to tamoxifen from E8.5. Scale bars, 50 µm.

e Contingency table showing the incidence of arrhythmic behavior in control (*Cdh5*^{CreERT2}neg-Rnf20$^{fl/fl}$) and *Rnf20*$^{iEC\text{-}KO}$ (*Cdh5*^{CreERT2}pos-Rnf20$^{fl/fl}$) hearts. Statistical significance was assessed using Fisher's exact test (p = 0.0011).

f Gross appearance of control (*Cdh5*^{CreERT2}neg-*Rnf20*$^{fl/fl}$) and *Rnf20*$^{iEC\text{-}KO}$ (*Cdh5*^{CreERT2}pos-*Rnf20*$^{fl/fl}$) embryos (left panels) and dissected hearts (right panels) at E12.5. Scale bars for left panels, 1 mm; Scale bars for right panels, 200 µm.

g TUNEL analysis performed on sections of control and *Rnf20*$^{iEC\text{-}KO}$ E11.5 embryos. Scale bars, 200 µm.

h, i Representative image of ECs morphology in P7 control and *Rnf20*^{iECKO} hearts (**h**) and quantification of normal versus abnormal EC morphology (arrows) normalized to the total EC count per field (**i**) (n=3 per group). Scale bars, 10 µm. Statistical significance was assessed using Fisher's exact test.

j Quantification of CM showing organized versus disorganized sarcomeres in P7 control (n=3) and *Rnf20*^{iECKO} (n=3) hearts normalized to the total number of CM per field. Statistical significance was assessed using Fisher's exact test.

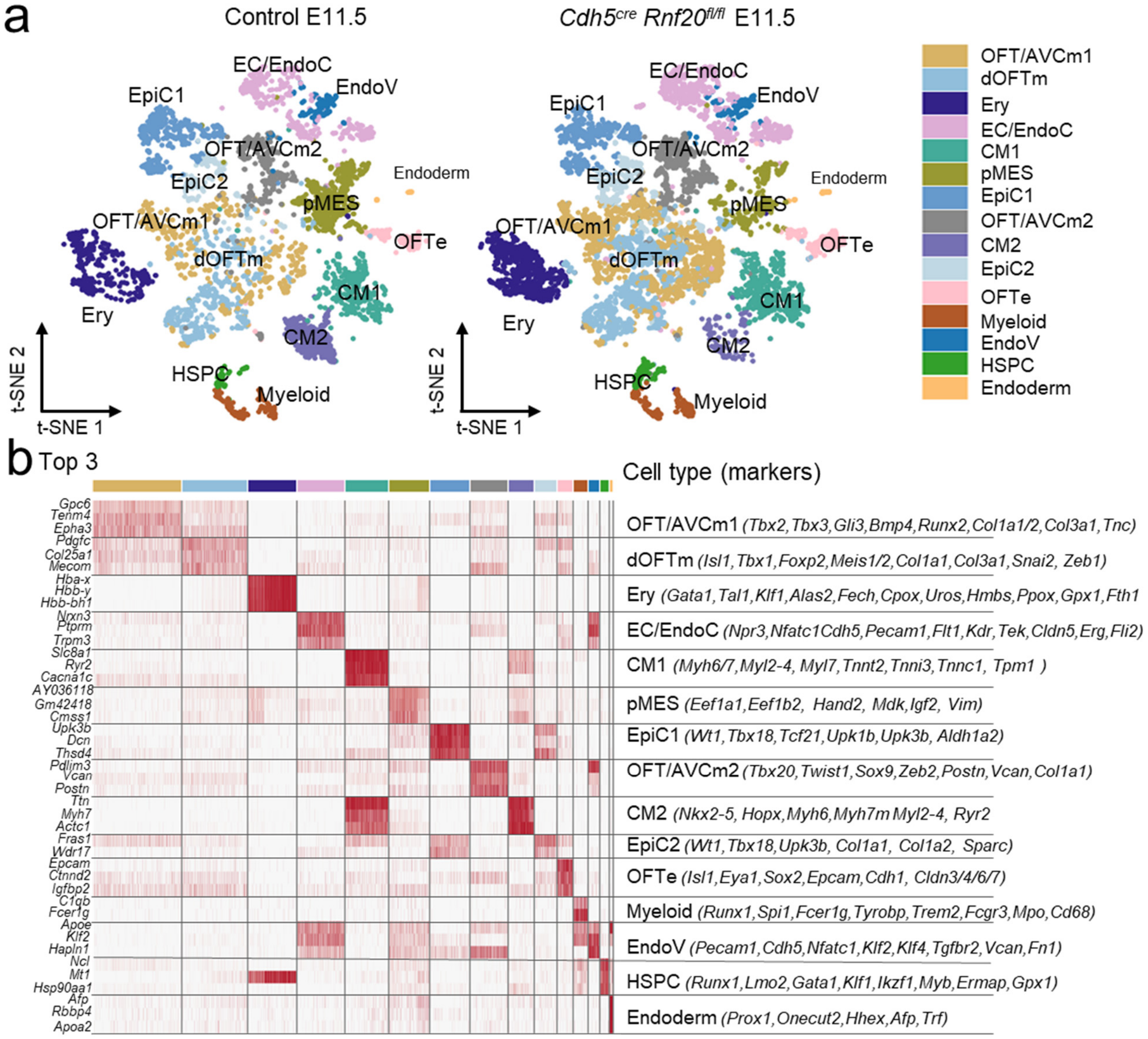

Supplementary Fig. 6. Single cell analysis of control and *Rnf20*$^{iEC-KO}$ hearts.

a t-SNE plot separated by genotype and colored according to cell type. **b** Heatmap showing the top 3 markers for each cell type on the left, and markers used to define the cell populations from Fig. 5b on the right.

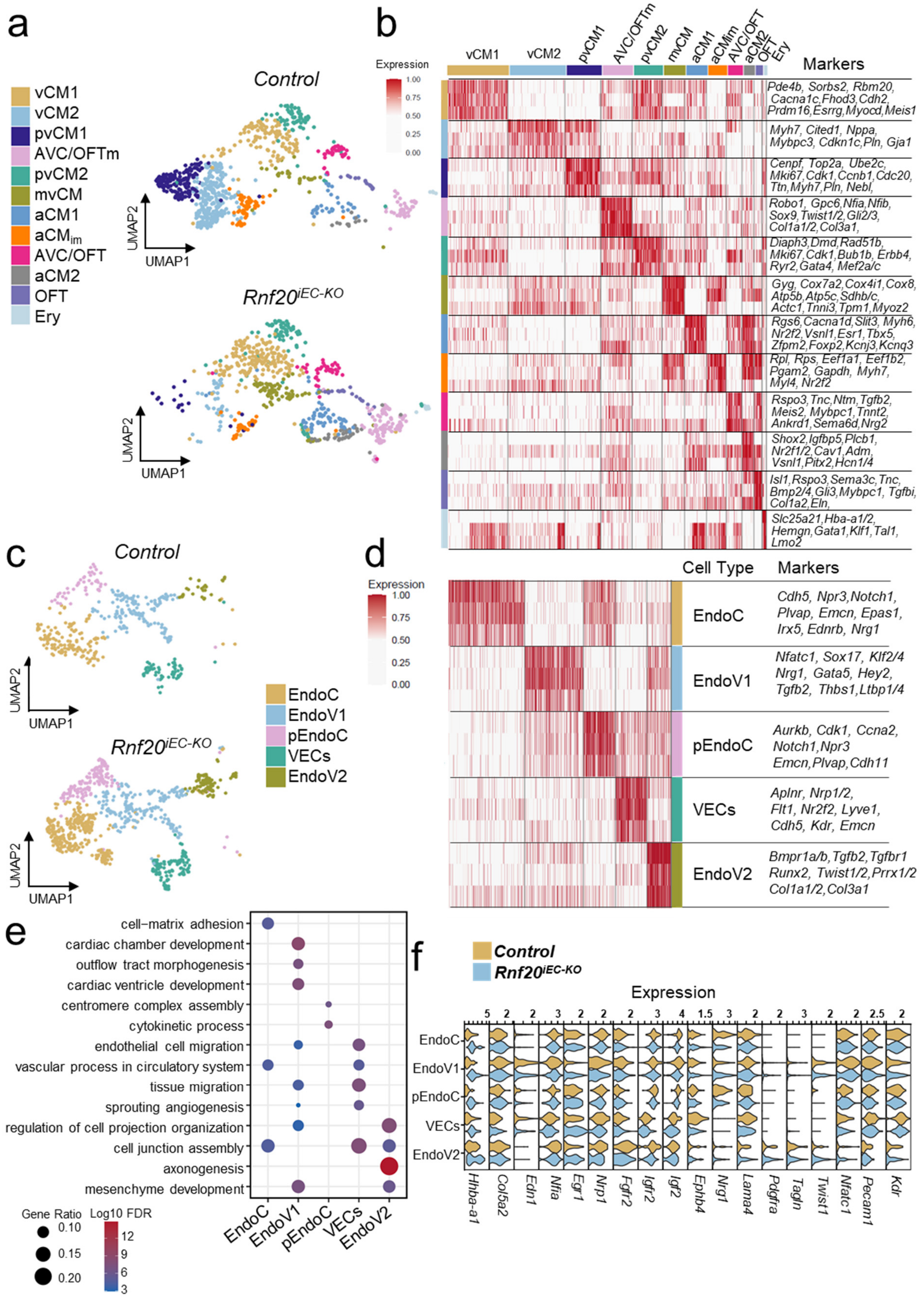
a
Control
Rnf20iEC-KO
UMAP1
UMAP2
vCM1
vCM2
pvCM1
AVC/OFTm
pvCM2
mvCM
aCM1
aCMim
AVC/OFT
aCM2
OFT
Ery
b
Expression
Markers
Pde4b, Sorbs2, Rbm20, Cacna1c, Fhod3, Cdh2, Prdm16, Esrrg, Myocd, Meis1
Myh7, Cited1, Nppa, Mybpc3, Cdkn1c, Pln, Gja1
Cenpf, Top2a, Ube2c, Mki67, Cdk1, Ccnb1, Cdc20, Ttn, Myh7, Pln, Nebl,
Robo1, Gpc6, Nfia, Nfib, Sox9, Twist1/2, Gli2/3, Col1a1/2, Col3a1,
Diaph3, Dmd, Rad51b, Mki67, Cdk1, Bub1b, Erbb4, Ryr2, Gata4, Mef2a/c
Gyg, Cox7a2, Cox4i1, Cox8, Atp5b, Atp5c, Sdhb/c, Actc1, Tnni3, Tpm1, Myoz2
Rgs6, Cacna1d, Slit3, Myh6, Nr2f2, Vsnl1, Esr1, Tbx5, Zfpm2, Foxp2, Kcnj3, Kcnq3
Rpl, Rps, Eef1a1, Eef1b2, Pgam2, Gapdh, Myh7, Myl4, Nr2f2
Rspo3, Tnc, Ntm, Tgfb2, Meis2, Mybpc1, Tnnt2, Ankrd1, Sema6d, Nrg2
Shox2, Igfbp5, Plcb1, Nr2f1/2, Cav1, Adm, Vsnl1, Pitx2, Hcn1/4
Isl1, Rspo3, Sema3c, Tnc, Bmp2/4, Gli3, Mybpc1, Tgfbi, Col1a2, Eln,
Slc25a21, Hba-a1/2, Hemgn, Gata1, Klf1, Tal1, Lmo2
c
Control
Rnf20iEC-KO
EndoC
EndoV1
pEndoC
VECs
EndoV2
d
Expression
Cell Type
Markers
EndoC
Cdh5, Npr3, Notch1, Plvap, Emcn, Epas1, Irx5, Ednrb, Nrg1
EndoV1
Nfatc1, Sox17, Klf2/4 Nrg1, Gata5, Hey2, Tgfb2, Thbs1, Ltbp1/4
pEndoC
Aurkb, Cdk1, Ccna2, Notch1, Npr3 Emcn, Plvap, Cdh11
VECs
Aplnr, Nrp1/2, Flt1, Nr2f2, Lyve1, Cdh5, Kdr, Emcn
EndoV2
Bmpr1a/b, Tgfb2, Tgfbr1 Runx2, Twist1/2, Prrx1/2 Col1a1/2, Col3a1
e
cell−matrix adhesion
cardiac chamber development
outflow tract morphogenesis
cardiac ventricle development
centromere complex assembly
cytokinetic process
endothelial cell migration
vascular process in circulatory system
tissue migration
sprouting angiogenesis
regulation of cell projection organization
cell junction assembly
axonogenesis
mesenchyme development
Gene Ratio
Log10 FDR
f
Control
Rnf20iEC-KO
Expression
Hhba-a1
Col5a2
Edn1
Nfia
Egr1
Nrp1
Fgfr2
Igfr2
Igf2
Ephb4
Nrg1
Lama4
Pdgfra
Tagln
Twist1
Nfatc1
Pecam1
Kdr

Supplementary Fig. 7. Cardiomyocyte and endothelial clusters in control and *Rnf20*$^{iEC-KO}$ hearts.

a t-SNE plot separated by genotype and colored according to CM subtype.
b Heatmap showing the top 3 markers for each CM subtype, and markers used to define the cell populations from Fig. 5e on the right.

c t-SNE plot separated by genotype and colored according to EC subtype.
d Heatmap showing the top 3 markers for each EC subtype, and markers used to define the cell populations from Fig. 5h on the right.

e Representative GO terms in the different EC populations. Dot size indicates the proportion of genes associated with given GO, while color represents the significance of enrichment.

f Violin plots visualizing expression levels of selected up- and downregulated genes in the different EC populations in control and *Rnf20*$^{iEC-KO}$ hearts. Values correspond to normalized gene expression counts across cell populations.

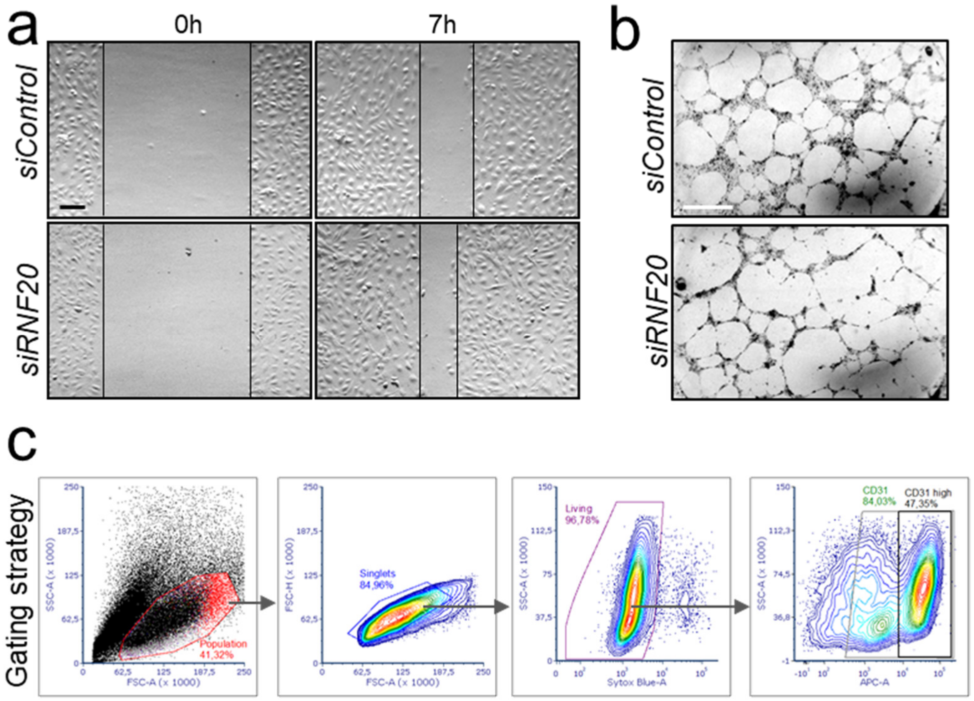

Supplementary Fig. 8. Impact of RNF20 loss on ECs.

a Representative images of a scratch assay with HUVECs transfected with control and siRNA against *RNF20* taken immediately after scratch (0h) and after 7h. Scale bars, 150 µm.

b Representative images of the tube formation assay with control and *siRNF20* transfected HUVECs after 24h. Scale bars, 10 µm.

c Gating strategy of the FACS sorting, illustrating sequential steps to exclude debris (FSC-A vs SSC-A), select singlets (FSC-A vs FSC-H), and identify viable cells (Sytox Blue–negative). Within the live singlet population, endothelial cells were defined as $CD31^{+}$ and $CD31^{high}$. Only the $CD31^{high}$ positive fraction was sorted for downstream applications.

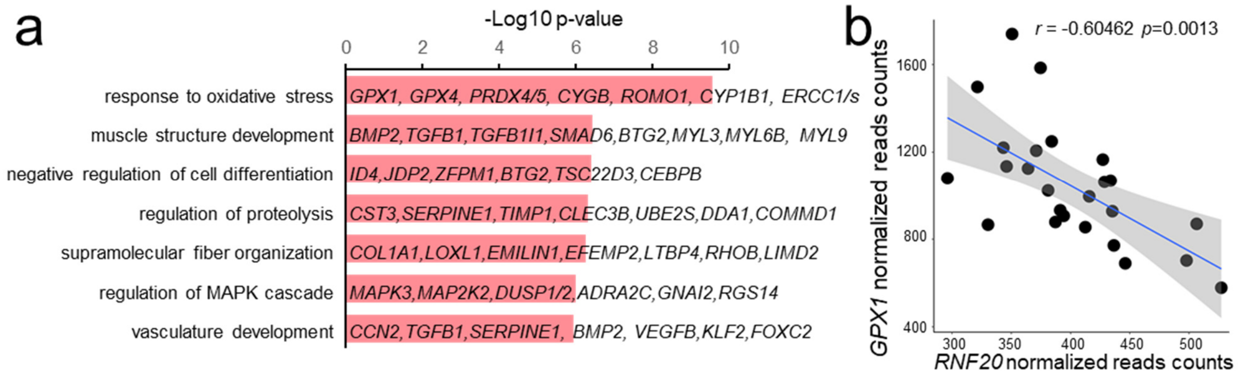

Supplementary Fig. 9. Genes negatively correlated with *RNF20* levels in TOF patients.

a GO analysis of genes exhibiting significant negative correlation (based on Spearman correlation) with *RNF20* levels. P-values were calculated using a hypergeometric test as performed by Metascape (v.3.5).

b Example showing significant negative correlation between *RNF20* levels and *GPX1* expression in samples from TOF patients. Each black dot represents an independent biological sample. The blue line corresponds to the linear regression fit, while the surrounding gray area denotes the 95% confidence interval, indicating the range within which the true regression line is likely to lie with 95% certainty. Spearman correlation was used to assess correlations between gene expression levels and clinical data, and *p*-values were estimated using a two-tailed test based on a *t*-distribution approximation.

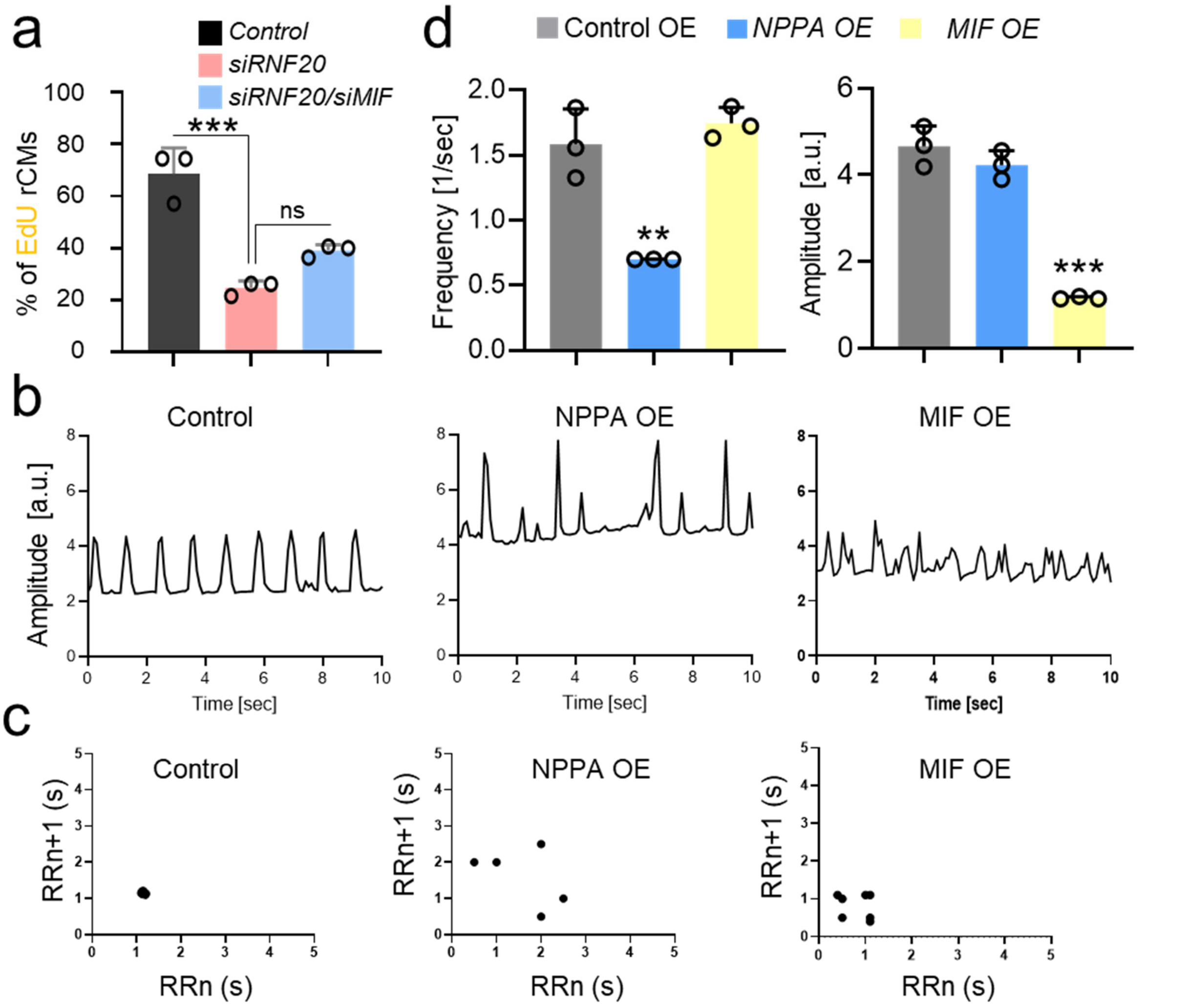

Supplementary Fig. 10. Effects of altered angiocrines on CM proliferation and contractility.

a Percentage of EdU-positive CMs in co-cultures with HUVECs transected with control siRNA (n=3), siRNA against *RNF20* (*siRNF20*, n=3), or *siRNF20* together with siRNAs against *MIF* (n=3), determined by EdU-incorporation for 24 hours.

b Plot of contraction amplitude and beating speed of rat CMs co-cultured with control HUVECs or HUVECs overexpressing NPPA and MIF extracted from video sequences using MYOCYTER.

c Poincaré plot derived from image sequences of beating rat CMs co-cultured with control HUVECs or HUVECs overexpressing NPPA and MIF.

d Frequency and amplitude of image sequences recorded on rCMs co-cultured with control HUVECs or HUVECs overexpressing NPPA and MIF.

Statistical analysis between multiple groups in **b**, was performed using one-way-ANOVA with Šídák's correction. Statistical analysis between control versus NPPA or MIF overexpressing group, in **d**, was performed using an unpaired two-tailed Student's t-test. Data are shown as means ± SEM. *p<0.05, **p<0.01, ***p<0.001.

Specimen's ID	Age	BSA	SpO2	RVP
2	96	0.31	95	77
5	116	0.33	94	70
6	120	0.3	70	80
7	120	0.27	72.5	77
8	162	0.33	92	77
9	164	0.32	NA	77
10	212	0.26	90	93
13	253	0.37	92	90
15	287	0.44	95	81
16	371	0.37	NA	72
17	385	0.42	96	80
18	406	0.48	95	91
20	727	0.54	87	80
22	224	0.27	84	50
23	134	0.26	95	18
27	253	0.32	95	84
28	208	0.23	75	60
29	119	0.22	87	60
32	51	0.21	78	43
37	195	0.33	NA	62
38	189	0.39	88	77
40	218	0.27	82	87
44	231	0.33	NA	60
48	194	0.35	88	72
49	245	0.41	99	95

Supplementary Table 1. Clinical features of TOF patients used in the study.

	SD1	SD2
Control1	0.059	0.037
Control2	0.0378	0.0327
Control3	0	0
Control4	0.0655	0.0824
Control5	0.071	0.085
Control6	0.137	0.079
Control7	0.107	0.065
Control8	0.0452	0.0452
Control9	0	0
Control10	0.071	0.071
Control11	0.156	0.145
Control12	0.0378	0.0535
$Isl1^{cre/+}Rnf20^{fl/fl}$ 1	0.093	0.038
$Isl1^{cre/+}Rnf20^{fl/fl}$ 2	0.113	0.07
$Isl1^{cre/+}Rnf20^{fl/fl}$ 3	0.417	0.372
$Isl1^{cre/+}Rnf20^{fl/fl}$ 4	0.522	0.412
$Isl1^{cre/+}Rnf20^{fl/fl}$ 5	0.159	0.254
$Isl1^{cre/+}Rnf20^{fl/fl}$ 6	0.141	0.282
	SD1	SD2
p value	0.0055	0.0013

Supplementary Table 2. SD1 and SD2 representing short- and long-term heart rate variability in Poincaré plots of control (n=12) and *$Isl1^{cre/+}Rnf20^{fl/fl}$* (n=6) hearts. Statistical analysis between two groups was performed using an unpaired two-tailed Student's t-test.

	SD1	SD2
Control1	0.0535	0.0378
Control2	0.15	0.106
Control3	0.093	0.035
Control4	0.0612	0.0306
Control5	0.0378	0.0327
Control6	0.0354	0.0306
Control7	0.0612	0.0354
$Cdh5^{CreERT2}$-$Rnf20^{fl/fl}$ 1	0.222	0.143
$Cdh5^{CreERT2}$-$Rnf20^{fl/fl}$ 2	0.519	0.412
$Cdh5^{CreERT2}$-$Rnf20^{fl/fl}$ 3	0.539	0.495
$Cdh5^{CreERT2}$-$Rnf20^{fl/fl}$ 4	0.339	0.465
$Cdh5^{CreERT2}$-$Rnf20^{fl/fl}$ 5	0.516	0.506
$Cdh5^{CreERT2}$-$Rnf20^{fl/fl}$ 6	0.417	0.358
$Cdh5^{CreERT2}$-$Rnf20^{fl/fl}$ 7	0.173	0.12
$Cdh5^{CreERT2}$-$Rnf20^{fl/fl}$ 8	0.787	0.546
$Cdh5^{CreERT2}$-$Rnf20^{fl/fl}$ 9	0.0378	0.0535
	SD1	SD2
p value	0.0024	0.001

Supplementary Table 3. SD1 and SD2 representing short- and long-term heart rate variability in Poincaré plots of control (n=7) and *Rnf20*$^{iEC\text{-}KO}$ (n=9) hearts. Statistical analysis between two groups was performed using an unpaired two-tailed Student's t-test.

	SD1	SD2
Control 1	0.118	0.121
Control 2	0.204	0.203
Control 3	0.173	0.113
$Cdh5^{CreERT2}$-$Rnf20^{fl/fl}$ 1	2.13	3.16
$Cdh5^{CreERT2}$-$Rnf20^{fl/fl}$ 2	1.479	1.152
$Cdh5^{CreERT2}$-$Rnf20^{fl/fl}$ 3	1.736	1.557
p value	0.0011	0,042

	SD1	SD2
siControl 1	0.173	0.171
siControl 2	0.048	0.032
siControl 3	0.123	0.131
siRNF20-1	0.921	0.615
siRNF20-2	0.697	0.813
siRNF20-3	0.554	0.488
RNF20 OE 1	0.0558	0.0373
RNF20 OE 2	0.0752	0.0824
RNF20 OE 3	0.0577	0.488

p value	SD1	SD2
siRNF20 vs siControl	0.0057	0.0069
RNF20 OE vs siControl	0.2327	0.5738

Supplementary Table 4. SD1 and SD2 representing short- and long-term beating rate variability in Poincaré plots of rat cardiomyocytes co-cultured with ECs isolated from control (n=3) and *Rnf20*$^{iEC-KO}$ (n=3) hearts or HUVECs following overexpression or silencing of RNF20 (n=3 for all groups). Statistical analysis between two groups was performed using an unpaired two-tailed Student's t-test.
