# Supplementary material for: Endothelial RNF20 suppresses endothelial-to-mesenchymal transition and safeguards physiological angiocrine signaling to prevent congenital heart disease
Source: Nat Commun. 2025 Oct 27;16:9480. doi: 10.1038/s41467-025-65291-0 (PMC12559710; doi:10.1038/s41467-025-65291-0)
Supplement: Supplementary file 3 — Description of Additional Supplementary Files [file 41467_2025_65291_MOESM3_ESM.pdf]

Description of Additional Supplementary Files

File Name: Supplementary Data 1
Description: Marker expression in different cardiac cell populations from control and Isl1cre/+Rnf20fl/fl embryos.

File Name: Supplementary Data 2
Description: DEG in single cell datasets of Isl1cre/+Rnf20fl/fl versus control embryos.

File Name: Supplementary Data 3
Description: Supplementary Data 3. Marker expression in different cardiac cell populations from control and Rnf20iEC-KO embryos.

File Name: Supplementary Data 4
Description: DEG in single cell datasets of Rnf20iEC-KO versus control embryos.

File Name: Supplementary Data 5
Description: DEG in RNA-seq datasets of sorted Pecam1^{+} shRnf20 KD ECs versus control differentiated ECs.

File Name: Supplementary Data 6
Description: Genes exhibiting significant negative correlation with RNF20 levels in TOF patients, based on Spearman correlation analysis. Listed are gene names, correlation coefficients, and associated p-value

File Name: Supplementary Data 7
Description: SD1 and SD2 represent short- and long-term beating rate variability in Poincaré plots of rat cardiomyocytes co-cultured with HUVECs following silencing with different siRNAs.

File Name: Supplementary Data 8
Description: Primers used in this study. qPCR primers were designed in-house using NCBI Primer-BLAST and synthesized by Sigma-Aldrich.
