# Supplementary material for: Endothelial RNF20 suppresses endothelial-to-mesenchymal transition and safeguards physiological angiocrine signaling to prevent congenital heart disease
Source: Nat Commun. 2025 Oct 27;16:9480. doi: 10.1038/s41467-025-65291-0 (PMC12559710; doi:10.1038/s41467-025-65291-0)
Supplement: Supplementary file 12 — Reporting Summary [file 41467_2025_65291_MOESM12_ESM.pdf]

nature portfolio

Corresponding author(s): Prof. Dr. Gergana Dobreva, Dr. Julio Cordero

Last updated by author(s): Sep 24, 2025

Reporting Summary

Nature Portfolio wishes to improve the reproducibility of the work that we publish. This form provides structure for consistency and transparency in reporting. For further information on Nature Portfolio policies, see our Editorial Policies and the Editorial Policy Checklist.

Statistics

For all statistical analyses, confirm that the following items are present in the figure legend, table legend, main text, or Methods section.

n/a	Confirmed	
☐	☒	The exact sample size (*n*) for each experimental group/condition, given as a discrete number and unit of measurement
☐	☒	A statement on whether measurements were taken from distinct samples or whether the same sample was measured repeatedly
☐	☒	The statistical test(s) used AND whether they are one- or two-sided<br>*Only common tests should be described solely by name; describe more complex techniques in the Methods section.*
☒	☐	A description of all covariates tested
☒	☐	A description of any assumptions or corrections, such as tests of normality and adjustment for multiple comparisons
☐	☒	A full description of the statistical parameters including central tendency (e.g. means) or other basic estimates (e.g. regression coefficient) AND variation (e.g. standard deviation) or associated estimates of uncertainty (e.g. confidence intervals)
☐	☒	For null hypothesis testing, the test statistic (e.g. *F*, *t*, *r*) with confidence intervals, effect sizes, degrees of freedom and *P* value noted<br>*Give P values as exact values whenever suitable.*
☒	☐	For Bayesian analysis, information on the choice of priors and Markov chain Monte Carlo settings
☒	☐	For hierarchical and complex designs, identification of the appropriate level for tests and full reporting of outcomes
☒	☐	Estimates of effect sizes (e.g. Cohen's *d*, Pearson's *r*), indicating how they were calculated

Our web collection on statistics for biologists contains articles on many of the points above.

Software and code

Policy information about availability of computer code

Data collection	The following standard software provided by instrument suppliers was used for data collection:<br>Histology: ZeissAxioScan(Zeiss)<br>Immunofluorescence/cryosection imaging: ZeissLSM710confocal microscope(Zeiss)<br>Bright field&live cell imaging(contractility, migration,EndMT, tube formation): LeicaDMI8inverted microscope(Leica)<br>Western blot: AmershamImager600(Cytiva)<br>FACS sorting/viability gating: BDFACSAria IIu cell sorter(BDBiosciences)<br>Chromatin shearing forChIP: Covarisultrasonicator<br>Single cell library prep: ChromiumController(10xGenomics) withNextGEM SingleCell3′Kitv3.1<br>Next generation sequencing: NextSeq550(Illumina) and BGISEQ 500platform(BGI)
Data analysis	Image&statistics:<br><br>ImageJ/Fiji1.53q with MYOCYTER v1.5 for contractility analysis<br>GraphPadPrism8 for plots & basic statistics<br><br>NGS analysis<br>Bowtie2 (v.2.4.4); https://bowtie-bio.sourceforge.net/bowtie2/index.shtml<br>ClusterProfiler (v.4.8.1); https://bioconductor.org/packages/release/bioc/html/clusterProfiler.html

Seurat (v.4.3.0); https://satijalab.org/seurat/articles/install.html
STAR (v. 2.7.10a); https://github.com/alexdobin/STAR
PIC repository; https://github.com/MiMiroot/PIC
rstatix (v. 0.7.2); https://rpkgs.datanovia.com/rstatix/
R (v4.3.0); https://cran.r-project.org/bin/windows/base/old/4.1.1/
NicheNet (v2.0.0); https://github.com/saeyslab/nichenetr
CellRanger (version7.1.0); https://www.10xgenomics.com/support/software/cell-ranger/latest/analysis/running-pipelines/cr-gex-count
BamTools version 2.5.1; https://github.com/pezmaster31/bamtools
EdgeR (version 3.42.4); https://bioconductor.org/packages/release/bioc/html/edgeR.html
DESeq2 (version 3.4.2); http://bioconductor.org/packages/release/bioc/vignettes/DESeq2/inst/doc/DESeq2.html
GraphPad Prism 8; https://www.graphpad.com/features
ngsplot (v. 2.47.1); https://github.com/shenlab-sinai/ngsplot
SAMtools version 1.7; http://www.htslib.org/
deepTools (v. 3.5.1); https://deeptools.readthedocs.io/en/develop/
HOMER (v. 5.1); http://homer.ucsd.edu/homer/
ggplot2 (v.3.4.2); https://ggplot2.tidyverse.org/

NGS visualization&browsing:
IGV2.7.0 for genome tracks
ImageJ/Fiji,GraphPadPrism,ggplot2(see above)

Code availability:
The full code is available at the indicated GitHub repository (https://github.com/jcorderJC12/03Rnf20_CHD_NatC) and additional information is available upon request to the corresponding authors.

For manuscripts utilizing custom algorithms or software that are central to the research but not yet described in published literature, software must be made available to editors and reviewers. We strongly encourage code deposition in a community repository (e.g. GitHub). See the Nature Portfolio guidelines for submitting code & software for further information.

Data

Policy information about availability of data

All manuscripts must include a data availability statement. This statement should provide the following information, where applicable:

- Accession codes, unique identifiers, or web links for publicly available datasets
- A description of any restrictions on data availability
- For clinical datasets or third party data, please ensure that the statement adheres to our policy

Chromatin immunoprecipitation (ChIP)-sequencing, bulk and single-cell RNA sequencing data generated in this study have been deposited in GEO, accession number GSE246928 [https://www.ncbi.nlm.nih.gov/geo/query/acc.cgi?acc=GSE246928]. Bulk RNA-seq of Tetralogy of Fallot (TOF) patients have been deposited in GEO, accession number GSE305562 [https://www.ncbi.nlm.nih.gov/geo/query/acc.cgi?acc=GSE305562]. Processed data are included in the Supplementary Data 1-8. RNA Polymerase II, H2Bub1 ChIP-seq and RNA-seq data from HUVECs have been previously deposited in GEO, accession number GSE212524 [https://www.ncbi.nlm.nih.gov/geo/query/acc.cgi?acc=GSE212524]. Single-cell RNA-seq data from Tetralogy of Fallot (TOF) patients and healthy donors were retrieved from GSE203274 [https://www.ncbi.nlm.nih.gov/geo/query/acc.cgi?acc=GSE203274]. Single-cell RNA-seq from E7.75 and E8.25 mouse embryos were retrieved from GSE108963 [https://www.ncbi.nlm.nih.gov/geo/query/acc.cgi?acc=GSE108963]. Source data are provided with this paper.

Research involving human participants, their data, or biological material

Policy information about studies with human participants or human data. See also policy information about sex, gender (identity/presentation), and sexual orientation and race, ethnicity and racism.

Reporting on sex and gender	Sex and gender were not considered in the study design or analysis.
Reporting on race, ethnicity, or other socially relevant groupings	Not applicable. No race, ethnicity, or other socially relevant variables were collected or analyzed in this study.
Population characteristics	Human data were obtained in accordance with the ethical approval from the Heidelberg Medical Faculty of the Heidelberg University (vote S-157/2013 and later amendments). No additional population characteristics were analyzed beyond the scope of the approved study.
Recruitment	Data were obtained under the approval of the Heidelberg Medical Faculty. The recruitment procedures were not conducted by the authors but followed the institutional and ethical regulations of the Heidelberg University.
Ethics oversight	All studies involving human subjects were conducted in accordance with the regulations of the Heidelberg Medical Faculty of the Heidelberg University, with ethical standards laid down in the vote S-157/2013 and its later amendments. Informed consents were obtained from parents/legally authorized representatives of participants.

Note that full information on the approval of the study protocol must also be provided in the manuscript.

Field-specific reporting

Please select the one below that is the best fit for your research. If you are not sure, read the appropriate sections before making your selection.

[x] Life sciences [ ] Behavioural & social sciences [ ] Ecological, evolutionary & environmental sciences

For a reference copy of the document with all sections, see nature.com/documents/nr-reporting-summary-flat.pdf

Life sciences study design

All studies must disclose on these points even when the disclosure is negative.

Sample size	No statistical measures were used to determine sample size. Sample size is based on our experience and on publications by other groups.
Data exclusions	None
Replication	All experiments were performed at least three independent times. All attempts at replication were successful.
Randomization	Mice were randomly allocated into experimental groups.
Blinding	The investigators were not blinded in regard to allocation of samples during experiments and outcome assessment. However, the outcomes were quantitative and not subjective.

Reporting for specific materials, systems and methods

We require information from authors about some types of materials, experimental systems and methods used in many studies. Here, indicate whether each material, system or method listed is relevant to your study. If you are not sure if a list item applies to your research, read the appropriate section before selecting a response.

Materials & experimental systems

n/a	Involved in the study	
[ ]	[x]	Antibodies
[ ]	[x]	Eukaryotic cell lines
[x]	[ ]	Palaeontology and archaeology
[ ]	[x]	Animals and other organisms
[ ]	[x]	Clinical data
[x]	[ ]	Dual use research of concern
[x]	[ ]	Plants

Methods

n/a	Involved in the study	
[x]	[ ]	ChIP-seq
[ ]	[x]	Flow cytometry
[x]	[ ]	MRI-based neuroimaging

Antibodies

Antibodies used

APC-conjugated CD31 (Pecam-1) antibody (eBioscience, #17031180)
p-SMAD2&p-SMAD3 primary antibody (1:100, Cell Signaling, D27F4)
DAPI (1:10000, Thermo Fisher Scientific)
anti-myosin heavy chain (MF20) (1:10, DSHB)
anti-phospho-Histone 3 (Serin 10) (Cell Signalling #9701S)
anti-Ubiquityl-Histone H2B (Lys120) (1:200, Cell Signaling, #5546)
Isolectin B4 (IB4) Alexa Fluor 647 (1:200, Thermo Fisher Scientific, #I32450)
WGA-alexa-fluor-633, (1:100, Invitrogen #W21404)
CD31 (1:100, BD #553370)
Troponin I (1:100, abcam, #ab56357)
SYTOX Blue (1:2000, Invitrogen, #S34857)
Donkey anti rabbit Alexa Fluor™ 488 –conjugated (1:400, Invitrogen, #A-21206)
Donkey anti Rat Alexa Fluor™ 633–conjugated (1:400, Invitrogen, #A-21094)
TCEA1 (Abcam, ab185947)

Validation

APC-conjugated CD31 (Pecam-1): https://www.thermofisher.com/antibody/product/CD31-PECAM-1-Monoclonal-Antibody-390-APC-eBioscience/17-0311-80
p-SMAD2&p-SMAD3 : https://www.cellsignal.com/products/primary-antibodies/phospho-smad2-ser465-467-smad3-ser423-425-d27f4-rabbit-mab/8828?srsltid=AfmBOoracRy3L771hPi9tRf10haD5KLpSYYgtoh7RC3m9aSvqoPSnGdY
DAPI : https://www.thermofisher.com/de/de/home/life-science/cell-analysis/fluorophores/dapi-stain.html

anti-myosin heavy chain (MF20) : https://dshb.biology.uiowa.edu/MF-20
anti-phospho-Histone H3 (Ser10) : https://www.cellsignal.com/products/primary-antibodies/phospho-histone-h3-ser10-antibody/9701?srsltid=AfmBOooidIlcfcMaGB-SCwftpPAHsz7Z3W-ZWqhB9Ei1qKaugprHbMEX
anti-Ubiquityl-Histone H2B (Lys120) : https://www.cellsignal.com/products/primary-antibodies/ubiquityl-histone-h2b-lys120-d11-xp-rabbit-mab/5546?srsltid=AfmBOori8RuwUtGs-D2tiCCODTzuRlOOqKHHql7U__qFmL6WaJlKx7bl
Isolectin B4 (IB4) Alexa Fluor 647 : https://www.thermofisher.com/order/catalog/product/de/en/I32450
WGA-alexa-fluor-633 : https://www.thermofisher.com/order/catalog/product/de/de/W21404
CD31 : https://www.bdbiosciences.com/en-eu/products/reagents/flow-cytometry-reagents/research-reagents/single-color-antibodies-ruo/purified-rat-anti-mouse-cd31.553370?tab=product_details
Troponin I: https://www.abcam.com/en-us/products/primary-antibodies/cardiac-troponin-i-antibody-ab56357
SYTOX Blue: https://www.thermofisher.com/order/catalog/product/de/de/S34857
Donkey anti rabbit Alexa Fluor™ 488 –conjugated : https://www.thermofisher.com/antibody/product/Donkey-anti-Rabbit-IgG-H-L-Highly-Cross-Adsorbed-Secondary-Antibody-Polyclonal/A-21206
Donkey anti Rat Alexa Fluor™ 633–conjugated : https://www.thermofisher.com/antibody/product/Goat-anti-Rat-IgG-H-L-Cross-Adsorbed-Secondary-Antibody-Polyclonal/A-21094
TCEA1 : https://www.abcam.com/en-us/products/primary-antibodies/tcea1-tcea2-tcea3-antibody-epr14821-ab185947?srsltid=AfmBOooxqnCuVqRmmt1McffcPO2MGC01dL8-3xNyBQIPWCSz51UQEiVd

Eukaryotic cell lines

Policy information about cell lines and Sex and Gender in Research

Cell line source(s)	HEK293T (American Type Culture Collection, Cat. no: CRL-3216)<br>HUVECs (human umbilical vein endothelial cells, pooled donors; PromoCell C-12203)<br>Rat CMs (from one to three day old postnatal rats) were isolated via Percoll-Gradient<br>mESCs (mouse ESCs) are from https://doi.org/10.1371/journal.pone.0002532
Authentication	HEK293T and HUVECs cells were authenticated by ATCC.
Mycoplasma contamination	Cell lines were mycoplasma negative.
Commonly misidentified lines (See ICLAC register)	No commonly misidentified lines were used in the study.

Animals and other research organisms

Policy information about studies involving animals; ARRIVE guidelines recommended for reporting animal research, and Sex and Gender in Research

Laboratory animals	Mice (Rnf20tm1a(EUCOMM)Wtsi, Isl1tm1(cre)Sev-C57BL/6, Cdh5CreERT2 mouse line Tg(Cdh5-cre/ERT2)1Rha) were housed in a pathogen-free animal facility under standard conditions with a 12 hour light/dark cycle, temperature of 20-25 degree and humidity range of 30-70%. All animal experiments were performed according to the institutional guidelines and are covered in an approved animal experimental protocolsby the Committee for Animal Rights Protection of the State of Baden-Württemberg (Regierungspraesidium Karlsruhe, Experimentalprotocol Az.: 35-9185.81/G-17/24).<br>The Rnf20tm1a(EUCOMM)Wtsi line was generated by microinjection of Rnf20 tm1a(EUCOMM)Wtsi ESCs, obtained from the European Conditional Mouse Mutagenesis Program (EUCOMM), into blastocysts, as detailed in ref. 41. For the generation of a conditional (floxed) allele Rnf20tm1c(EUCOMM)Wtsi line, the Rnf20tm1a(EUCOMM)Wtsi mouse line was crossed with germline FLP deleter mouse line. Cre deleter lines were bred to Rnf20tm1c(EUCOMM)Wtsi mice to induce specific deletion of Rnf20 in Isl1-positive, Tie2-positive and Cdh5-positive cells following the presented in the figures experimental schemes.<br>Isl1Cre mouse line (Isl1tm1(cre)Sev-C57BL/6) was a kind gift from Sylvia Evans. The Tg(Tek-cre)12Flv line was obtained from Jackson Laboratory. The Cdh5CreERT2 mouse line Tg(Cdh5-cre/ERT2)1Rha was obtained from Prof. Ralf Adams. All mice used in this study were maintained on a C57BL/6 background.
Wild animals	No wild animals were used in this study.
Reporting on sex	Both male and female mice were used within the study.
Field-collected samples	No field-collected samples were used in this study.
Ethics oversight	All animal experiments were performed according to the regulations issued by the Committee for Animal Rights Protection of the State of Hessen (Regierungspraesidium Darmstadt, Germany) and the Committee for Animal Rights Protection of the State of Baden-Württemberg (Regierungspraesidium Karlsruhe, Experimentalprotocol Az.: 35-9185.81/G-17/24).

Note that full information on the approval of the study protocol must also be provided in the manuscript.

Clinical data

Policy information about clinical studies

All manuscripts should comply with the ICMJE guidelines for publication of clinical research and a completed CONSORT checklist must be included with all submissions.

Clinical trial registration	Not applicable. This study did not involve a registered clinical trial.
Study protocol	All studies involving human subjects were conducted in accordance with the regulations of the Heidelberg Medical Faculty of the Heidelberg University, with ethical standards laid down in the vote S-157/2013 and its later amendments.
Data collection	Human data were obtained under the approval of the Heidelberg Medical Faculty. Data collection was performed at Heidelberg University following institutional and ethical regulations. Recruitment and data acquisition were not conducted by the authors but were performed within the approved framework.
Outcomes	This study did not involve predefined clinical outcomes. The analyses were focused on mechanistic and molecular aspects derived from the approved human data.

Plants

Seed stocks	*Report on the source of all seed stocks or other plant material used. If applicable, state the seed stock centre and catalogue number. If plant specimens were collected from the field, describe the collection location, date and sampling procedures.*
Novel plant genotypes	*Describe the methods by which all novel plant genotypes were produced. This includes those generated by transgenic approaches, gene editing, chemical/radiation-based mutagenesis and hybridization. For transgenic lines, describe the transformation method, the number of independent lines analyzed and the generation upon which experiments were performed. For gene-edited lines, describe the editor used, the endogenous sequence targeted for editing, the targeting guide RNA sequence (if applicable) and how the editor was applied.*
Authentication	*Describe any authentication procedures for each seed stock used or novel genotype generated. Describe any experiments used to assess the effect of a mutation and, where applicable, how potential secondary effects (e.g. second site T-DNA insertions, mosiacism, off-target gene editing) were examined.*

Flow Cytometry

Plots

Confirm that:

- [x] The axis labels state the marker and fluorochrome used (e.g. CD4-FITC).
- [x] The axis scales are clearly visible. Include numbers along axes only for bottom left plot of group (a 'group' is an analysis of identical markers).
- [x] All plots are contour plots with outliers or pseudocolor plots.
- [x] A numerical value for number of cells or percentage (with statistics) is provided.

Methodology

Sample preparation	mESC-derived EC (day 7) were enzymatically dissociated, washed with PBS, and subsequently blocked in FACS buffer (PBS supplemented with 0.4% BSA) for 5 minutes at ambient temperature. Cells were stained with APC-conjugated CD31 (Pecam-1) antibody (eBioscience, #17031180) for EC identification. Cells were washed twice with FACS buffer followed by Sytox Blue (1:2000, Invitrogen, # S34857) staining before flow cytometric data acquisition.
Instrument	BD FACSAria IIu instrument equipped with Violet (405nm), Blue (488nm), Yellow-Green (561nm) and Red (640nm) Laser
Software	BD FACS Canto Software; FCS Express 7
Cell population abundance	EC were identified as SytoxBlue negative and CD31 positive Population and represnted aproximately 75% of the population.
Gating strategy	Cells were sequentially gated to exclude debris (FSC-A vs SSC-A), select singlets (FSC-A vs FSC-H), and identify viable cells (Sytox Blue–negative). Within the live singlet population, endothelial cells were defined as CD31+ and CD31high. Only the CD31high fraction was sorted for downstream applications.

- [x] Tick this box to confirm that a figure exemplifying the gating strategy is provided in the Supplementary Information.
